# Multiple micronutrient supplementation improves vitamin B_12 _and folate concentrations of HIV infected children in Uganda: a randomized controlled trial

**DOI:** 10.1186/1475-2891-10-56

**Published:** 2011-05-21

**Authors:** Grace Ndeezi, James K Tumwine, Christopher M Ndugwa, Bjørn J Bolann, Thorkild Tylleskär

**Affiliations:** 1 Department of Paediatrics and Child Health, School of Medicine, College of Health Sciences, Makerere University, Kampala, Uganda; 2 Centre for International Health, University of Bergen, Norway; 3 Institute of Medicine, University of Bergen, and Laboratory of Clinical Biochemistry, Haukeland University Hospital, Bergen, Norway

## Abstract

**Background:**

The effect of multiple micronutrient supplementation on vitamin B_12 _and folate has hither to not been reported in African HIV infected children. This paper describes vitamin B_12 _and folate status of Ugandan HIV infected children aged 1-5 years and reports the effect of multiple micronutrient supplementation on serum vitamin B_12 _and folate concentrations.

**Methods:**

Of 847 children who participated in a multiple micronutrient supplementation trial, 214 were assessed for vitamin B_12 _and folate concentrations pre and post supplementation. One hundred and four children were randomised to two times the recommended dietary allowance (RDA) of a 14 multiple micronutrient supplement (MMS) and 114 to a 'standard of care' supplement of 6 multivitamins (MV). Serum vitamin B_12 _was measured by an electrochemiluminescence immunoassay and folate by a competitive protein-binding assay using Modular E (Roche) automatic analyzer. Vitamin B_12 _concentrations were considered low if less than 221picomoles per litre (pmol/L) and folate if < 13.4 nanomoles per litre (nmol/L). The Wilcoxon Signed Ranks test was used to measure the difference between pre and post supplementation concentrations.

**Results:**

Vitamin B_12 _was low in 60/214 (28%) and folate in 62/214 (29.0%) children. In the MMS group, the median concentration (IQR) of vitamin B_12 _at 6 months was 401.5 (264.3 - 518.8) pmol/L compared to the baseline of 285.5 (216.5 - 371.8) pmol/L, p < 0.001. The median (IQR) folate concentrations increased from 17.3 (13.5 - 26.6) nmol/L to 27.7 (21.1 - 33.4) nmol/L, p < 0.001. In the 'standard of care' MV supplemented group, the median concentration (IQR) of vitamin B_12 _at 6 months was 288.5 (198.8 - 391.0) pmol/L compared to the baseline of 280.0 (211.5 - 386.3) pmol/L while the median (IQR) folate concentrations at 6 months were 16.5 (11.7 - 22.1) nmol/L compared to 15.7 (11.9 - 22.1) nmol/L at baseline. There was a significant difference in the MMS group in both vitamin B_12 _and folate concentrations but no difference in the MV group.

**Conclusions:**

Almost a third of the HIV infected Ugandan children aged 1-5 years had low serum concentrations of vitamin B_12 _and folate. Multiple micronutrient supplementation compared to the 'standard of care' supplement of 6 multivitamins improved the vitamin B_12 _and folate status of HIV infected children in Uganda.

**Trial registration:**

http://ClinicalTrials.govNCT00122941)

## Background

Vitamin B_12 _and folate deficiency are relatively common in low income countries compared to high income countries, particularly in communities where the diet is predominantly vegetarian [1-4], where major sources of vitamin B_12 _such as meat, fish, poultry, milk and fortified breakfast cereals [5] are consumed in small amounts or are not readily available. Dietary sources of folate, on the other hand, are more prevalent as they include green leafy vegetables, fruits and dried beans and peas [6]. During infancy and childhood folate and vitamin B_12 _deficiency have been associated with failure to thrive, reduced physical activity and cognitive function and megaloblastic anaemia [7,8]. However many of the symptoms are non-specific and may result from a variety of medical conditions.

A significant number of HIV-infected children in low-income countries remain at risk of increased morbidity due to immunodeficiency related to HIV infection in addition to micronutrient deficiencies. Low vitamin B_12 _status has been associated with poor immunological status and HIV disease progression in adults [9]. In follow up studies of HIV infected pregnant women micronutrient supplementation resulted in improved child growth, haematological indices and vitamin B_12 _levels [10].

Food supplementation and multiple micronutrient fortification of foods and beverages in children with unknown HIV status living in low income countries have shown improved haemoglobin levels and concentrations of deficient micronutrients [2,11-14].

We hypothesised that supplementation with two recommended dietary allowances (RDA) of 14 multiple micronutrients (MMS) would increase serum vitamin B_12 _and folate concentrations compared to a 1 RDA of 6 multivitamin (MV) 'standard of care' supplement. We here report the baseline vitamin B_12 _and folate status in Ugandan HIV infected children aged 1-5 years and the effect of multiple micronutrient supplementation on serum concentrations of vitamin B_12 _and folate.

## Methods

### Design

The study was a randomised controlled trial conducted between 2005 and 2008 at 7 paediatric HIV clinics in Uganda. Participants were allocated to the intervention or 'standard of care' supplement in a 1:1 ratio.

### Participants

HIV infected children aged 1- 5 years presenting at the study clinics for follow up visits were eligible for the trial. Children who had enrolled in other studies or were unable to adhere to a regular follow up schedule and those whose mothers or guardians declined consent were excluded. There were no other micronutrient supplementation studies going on at the time so the exclusion for those who were participating in other studies was done to avoid participant fatigue and interference with procedures. Multivitamin supplementation was routinely practiced at the study clinics but this was not an exclusion criteria. Eligible participants were consecutively enrolled, assigned to the study intervention or 'standard of care' supplement and followed for one year.

The study sites have been previously described [15]. Three sites were located in the capital city of Uganda (Kampala) and these were Mulago (the national referral hospital), Mildmay Centre and Nsambya hospital. The other sites were situated in the regional hospitals in the east, south-west, central and north of the country. For logistical reasons, transport and storage, it was not possible to collect samples for biochemical tests from all the sites. Therefore blood samples for biochemical tests were only collected from the three Kampala sites. The samples were subsequently shipped to the clinical chemistry laboratory at Haukeland University Hospital, Bergen (Norway), where vitamin B_12 _and folate were analysed.

The trial enrolled 847 children at all the seven study sites. Out of 705 children from whom blood samples could be stored for micronutrient tests, 261 had no sufficient samples, 230 had other micronutrient tests done or had either baseline but no result at the second sampling. Because of multiple analyses 214 children had both baseline and 6 months results for vitamin B_12 _and folate concentrations. There were no significant differences in demographic and clinical characteristics such as age, sex, anthropometric measurements and other laboratory measurements like CD4 + cell count among those who had results for vitamin B_12 _and folate compared to those who did not.

The study was approved by the College of Health Sciences Research and Ethics Committee, Makerere University, Kampala, Uganda; the Uganda National Council for Science and Technology, and the Regional Committee for Medical Research Ethics, Western Norway.

Cotrimoxazole prophylaxis, initiation of ART, management of common illnesses and opportunistic infections was offered using the national guidelines for Paediatric HIV care.

### Intervention

The trial supplements were manufactured in powder form and packaged by NUTRISET, France using a formula that was determined by the investigators based on twice the recommended dietary intake for a 4 year old category [6]. We decided to use 2 RDA based on the fact that many children were malnourished despite routine supplementation with multivitamins. Secondly some previous studies of HIV infected adults had indicated that HIV infected persons may require multiples of RDA in order to achieve normal serum concentrations of micronutrients. Thirdly, looking at the tables of the nutritional requirements for children [6] we noted that there are 2 age bands where our participants belonged, 1-3 and 4-8 years of age. We noticed that there were minor variations in dosages of some micronutrients while others like iodine and vitamin D did not vary. We therefore decided to use the 4 year old category for our study. In addition it was easier to administer a uniform intervention. There were no similar studies in the region and there was no literature to guide us on the micronutrient status of Ugandan HIV infected children. Also there were no food composition tables based on the local foods so we could not estimate how much micronutrients children get from the usual diet. The MMS comprised of 800 mcg vitamin A, 1.2 mg vitamin B_1_, 1.2 mg vitamin B_2_, 16 mg niacin, 1.2 mg vitamin B_6_, 2.4 mcg vitamin B_12_, 50 mg vitamin C, 400 IU vitamin D, 14 mg vitamin E, 40 mcg folate, 60 mcg selenium, 10 mg zinc, 800 mcg copper and 180 mcg iodine. The multivitamin 'standard of care' supplement contained 400 mcg vitamin A, 0.6 mg vitamin B_1_, 0.6 mg vitamin B_2_, 8 mg niacin, 25 mg vitamin C and 200 IU vitamin D. The contents and formula for the MV supplement was based on the regular multivitamins supplied at the study clinics as routine care of HIV infected children. The daily dose was 4 g and this was equivalent to a levelled scoop supplied by the manufacturer. The powder was mixed with 10 to 20 ml of milk or water. The first dose of the supplement was administered at the study clinic following a demonstration and under observation by the study nurse. At the time of administering the first dose we counselled the mother on the importance of completing the dose and ensuring that the daily dose was given. Whenever the child vomited during or within 30 minutes of administering the dose a repeat dose was given. Mothers were given calendar charts and instructed to tick on the appropriate date whenever a dose was given. They would return to the clinic with the container/remaining supplement together with the calendar charts on routine follow up visits. The remaining amount of supplement was measured using a light weight scale and the level of compliance determined using the proportion of the supplement consumed against the expected. The supply for one month was 140 grams and the expected dose for 30 days was 120 grams. The remaining 20 grams was to cater for vomited doses or in case there was spillage. The mothers administered the subsequent doses from home and it was not possible to observe them. The supplements were given for a period of 6 months when the second sample of blood was drawn.

### Outcomes

The outcome measures were serum vitamin B_12 _and folate concentrations pre and post supplementation and factors associated with low concentrations at baseline. These were secondary objectives of the trial. Survival was assessed as the primary objective whose findings have been previously reported [15].

### Randomisation and blinding

The randomisation sequence was generated using the stata soft ware in variable blocks of 4 to 20. The supplements were manufactured and packaged in 140 g plastic containers which were sequentially numbered. RB generated the randomisation sequence at Geneva and sent it to the manufacturers in France. The randomisation code was kept at Geneva and by the manufacturers. The treatment assignment was revealed upon completion of the study.

Participants were enrolled by the principal investigator and the other study doctors. The trial nurse at the study sites dispensed the supplement in serial order. The colour, consistency and odour of the intervention and 'standard of care' supplement were similar. The principal investigator, study personnel including doctors and nurses and the caretakers/participants were all blinded to treatment assignment.

### Clinical and laboratory measurements

At enrolment demographic information, history of the child's illness and findings on physical examination were recorded. Non fasting blood samples were drawn for CD4+ cell count and micronutrient analysis using procedures previously described in the same cohort [15]. Vitamin B_12 _was measured by electrochemiluminescence immunoassay, and folate by a competitive protein-binding assay on Modular E (Roche) automatic analyzer. The coefficient of variation for the assays was less than 5%. To determine cut off values for low vitamin B_12 _and folate, we considered what other researchers have used since there were no reference values for Ugandan children. Vitamin B_12 _concentrations were considered low if less than 221 picomoles per litre (pmol/L), combining both very low (<148 pmol/L) and marginal (148 - 221 pmol/L) status. Concentrations equal or > 221 pmol/L was considered to be normal. Folate concentrations were low if < 13.4 nanomoles per litre (nmol/L), similarly combining both very low (<6.8 nmol/L) and marginal (6.8 - 13.4 nmol/L) status. Concentrations ≥ 13.4 nmol/L were regarded as being normal [16,17].

### Statistical analysis

Change in micronutrient concentrations of each participant was computed in SPSS as follows: concentration at 6 months minus concentration at baseline. Medians and their interquartile ranges were used for summarising the data. Because vitamin B_12 _and folate concentrations were not normally distributed the Wilcoxon Signed Ranks test was used to measure the difference between baseline and 6 months in each group. Differences in categorical variables were tested with the Pearson chi-square or Fischer's exact test. Differences were considered significant if a two-sided p-value was less than 0.05. Multiple regression analysis was performed to examine factors associated with low vitamin B_12 _or folate concentrations at baseline assessment.

## Results

### Baseline characteristics of participants

**Figure 1:**
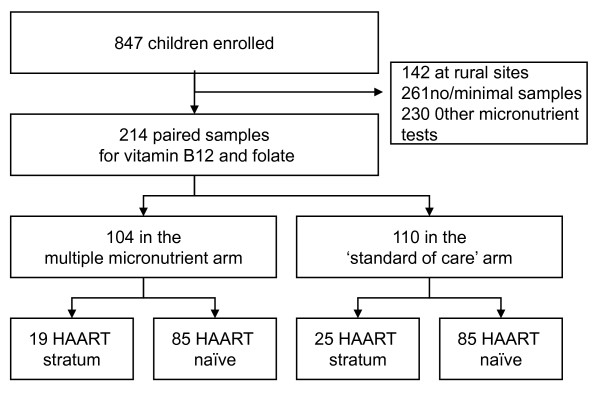
**Study profile**.

Of the 214 children with both baseline and 6 months results for vitamin B_12 _and folate, 104 (48.6%) received MMS while the rest received the 'standard of care' MV supplement. The distribution of children by arm and strata is presented in figure 1. Males and females were equally represented. The median age (IQR) was 33.2 months (19.6 - 44.4) in the MMS and 30.3 (19.8 - 45.5) in the MV group. Baseline characteristics are described in table 1. There were no significant differences between the two treatment groups.

**Table 1:** Characteristics of 214 Ugandan HIV infected children analysed for vitamin B_12 _and folate by intervention group

	Number (N)	MMS n (%)	'Standard of care' MV n (%)	p-value
Sex: Male	100	49 (49.0)	51 (51.0)	>0.99
Age less than 24 months	70	33 (47.1)	37 (52.9)	0.77
Mother as carer	146	73 (50.0)	73 (50.0)	0.56
On HAART	44	19 (43.2)	25 (56.8)	0.50
Routine CTX	193	93 (48.2)	100 (51.8)	0.82
Routine MV	144	68 (47.2)	76 (52.8)	0.66
WHZ < -2	28	14 (50.0)	14 (50.0)	>0.99
HAZ < -2	113	56 (49.6)	57 (50.4)	0.89
WHO stage 3 or 4	56	26 (46.4)	30 (53.6)	0.76
CD4+ T cells < 25%	114	50 (43.9)	64 (56.1)	0.20
Elevated CRP (> 6 g/dl)	67	33 (49.3)	34 (50.7)	0.42
Low haemoglobin (< 11 g/dl)	127	61 (48.0)	66 (52.0)	0.89
Low vitamin B_12 _(< 221 pmol/L)	60	29 (48.3)	31 (51.7)	0.96
Low folate (< 13.4 nmol/L)	62	25 (40.3)	37 (59.7)	0.13

MMS = multiple micronutrient supplementation

HAART = Highly active anti-retroviral therapy

CTX = Cotrimoxazole

MV = 'standard of care' multivitamins

WHZ = Weight for height z-score

HAZ = Height for age z-score

WHO = World Health Organisation

CD4+ = Cluster of differentiation 4

CRP = C-reactive protein

Overall, 60 children (28.0%) had low vitamin B_12 _concentrations less than 221.0 pmol/L, and 62 (29.0%) had low folate concentrations less than 13.4 nmol/L. Among the children with low vitamin B_12 _status, 13 (6.0%) had very low and 47 (22.0%) marginal concentrations (148 - 221 pmol/L). Of the 62 children with low folate status, 3 (1.4%) had very low (<6.8 nmol/L) and 59 (27.6%) had marginal concentrations (6.8 - 13.4 nmol/L).

### Comparisons between baseline and 6 months' vitamin B_12 _and folate concentrations

Baseline median serum vitamin B_12 _and folate concentrations were similar in the two groups. Following supplementation with multiple micronutrients (MMS) vitamin B_12 _concentrations increased from a median (IQR) of 285.5 (216.5 - 371.8) to 401.5 (264.3 - 518.8) pmol/L at 6 months. This difference was statistically significant (p < 0.001). Similarly folate concentrations increased from 17.3 (13.5 - 26.6) to 27.7 (21.1 - 33.4) nmol/L and this difference was also significant, p < 0.001 (Table 2). There was no significant difference in the MV 'standard of care' group.

**Table 2:** Biochemical and haematological measurements at baseline and at 6 months of supplementation by intervention group

	Multiple Micronutrient Supplementation group (n = 104)		Comparative 'standard of care' multivitamins group (n = 110)	
Measurement	Median (IQR)	P-value	Median (IQR)	p-value
Vitamin B_12 _(pmol/L)				
Baseline	285.5 (216.5 - 371.8)	<0.001	280.0 (211.5 - 386.3)	0.78
6 months	401.5 (264.3 - 518.8)		288.5 (198.8 - 391.0)	
Change	90.5 (-0.8 - 203.5)		10.0 (-73.8 - 83.8)	
Folate (nmol/L)				
Baseline	17.3 (13.5 - 26.6)	<0.001	15.7 (11.9 - 22.1)	0.44
6 months	27.7 (21.1 - 33.4)		16.5 (11.7 - 22.1)	
Change	8.0 (-0.3 - 17.1)		-0.6 (-3.5 - 5.8)	
Haemoglobin (g/dl)				
Baseline	10.0 (8.7 - 11.2)	0.04	9.8 (8.8 - 11.2)	<0.001
6 months	10.9 (9.4 - 11.7)		10.6 (9.6 - 11.7)	
Change	0.3 (-0.4 - 0.9)		0.6 (-0.2 - 1.4)	
CD4+ count (cells/μL)				
Baseline	1201 (822 - 1556)	0.16	1033 (728 - 1406)	0.52
6 months	1039 (725 - 1358)		1043 (704 - 1484)	
Change	-137 (-348 - 254)		35 (-278 - 352)	

Wilcoxon Signed Ranks test was used to measure the difference between baseline and 6 months.

Of the 44 children who were on HAART, 19 received MMS while 25 received MV. In this stratum the median (IQR) concentration of vitamin B_12 _in the MMS arm at baseline was 262.0 (215.0 - 342.0) compared to 453.0 (261.0 - 594.0) pmol/L at 6 months, p = 0.002. The median (IQR) folate concentrations increased from 18.6 (13.8 - 23.9) to 25.0 (21.3 - 32.9) nmol/L, p = 0.040. Although the numbers were small these were significant differences. There was no significant difference in the MV group. Median (IQR) baseline vitamin B_12 _concentrations were higher in the HAART stratum [306.5 (209.3 - 372.8) pmol/L] compared to the non-HAART stratum [280.0 (218.0 - 386.3) pmol/L], but not significantly so. Folate concentrations were almost the same [16.1 (13.5 - 22.0) nmol/L] in the HAART and [16.0 (12.1 - 24.9) nmol/L] non-HAART stratum. Thirteen out of 40 children (32.5%) who were on HAART had CD4+ cell percent < 25 compared to 100/159 (63.5%) who were HAART naïve, p = 0.001 (Fisher's exact test). Median (IQR) duration of anti-retroviral therapy was 9 (6.0 - 14.3) months.

### Vitamin B_12 _and folate status at 6 months of supplementation

Overall, 42 (19.6%) children had low concentrations of vitamin B_12 _at 6 months, 9 (4.2%) with very low and 33 (15.4%) with marginal concentrations. Nine children (1.4%) were in the MMS and 33 (78.6%) in the MV group. This was a statistically significant difference. The odds ratio was 4.5 (95% CI; 2.0 - 10.0). Folate concentrations were low in 44/214 (20.6%) children at 6 months of follow up; almost all of them had marginal concentrations. Five children (11.4%) were supplemented with MMS and 39 (88.6%) MV; Odds ratio 10.8 (95% CI; 4.1 - 28.9). Low vitamin B_12 _and folate concentrations were more frequent in the MV supplemented group.

### Other clinical and haematological findings

Eight of the 214 children had signs of neurological disease, 6 with delayed or loss of developmental milestones, 3 of whom had low or marginal vitamin B_12 _status. There was a significant increase in the haemoglobin status in both groups as shown in table 2. There was no significant change in the CD4 cell counts in either the MMS or MV group.

### HAART strata and initiation of HAART during the study

Of the 44 children on HAART at enrolment, 15 (34.1%) and 10 (22.7%) had low vitamin B_12 _and low folate concentrations, respectively. The prevalence of low micronutrient status of these two was not significantly different between the HAART and non-HAART treated children. Of the 170 HAART naïve children, 21 started HAART during the study; 13 in MMS and 8 in MV group. This difference was not significant, p = 0.35. Seven of the 21 children who initiated HAART during the study had low vitamin B_12 _concentrations at enrolment.

### Factors associated with low vitamin B_12 _or folate concentrations

Vitamin B_12 _status was not closely associated with most of the baseline characteristics (Table 3). However being male, age less than 24 months and a haemoglobin < 11 g/dl were associated with low folate concentrations. At multivariate analysis only age and the male sex remained significantly associated with low serum folate concentrations.

**Table 3:** Factors associated with low vitamin B_12 _and folate among HIV infected children in Uganda

Baseline characteristics	Number N	**Low vitamin B**_**12 **_**status**^**a **^**n (%)**	Un adjusted OR (95% CI)	Adjusted OR (95%CI)
**Vitamin B**_**12 **_**status**				
Age < 24 months	70	19 (27.1)	0.9 (0.6 - 1.5)	
Age ≥ 24 months	144	41 (28.5)		
Male	100	33 (33.0)	1.4 (0.9 - 2.1)	1.6 (0.8 - 3.1)
Female	114	27 (23.7)		
Weight for height *z *score < -2	28	7 (25.0)	0.9 (0.4 - 1.7)	
Weight for height *z *score ≥ -2	182	52 (28.6)		
Haemoglobin < 11 g/dl	127	37 (29.1)	1.1 (0.7 - 1.7)	
Haemoglobin ≥ 11 g/dl	87	23 (26.4)		
CD4+ < 25%	114	33 (28.9)	1.1 (0.6 - 2.1)	
CD4+ ≥ 25%	85	23 (27.1)		
On HAART	44	15 (34.1)	1.3 (0.8 - 2.1)	
HAART naïve	170	45 (26.5)		
Routine CTX prophylaxis	193	56 (29.0)	1.5 (0.6 - 3.8)	
No routine CTX prophylaxis	21	4 (19.0)		
Previous routine multivitamins	144	40 (27.8)	1.0 (0.6 - 1.5)	
No routine multivitamins	70	20 (28.6)		
				
**Folate status**		**Low folate**		
Age < 24 months	70	27 (38.6)	1.6 (1.1 - 2.4)	2.2 (1.1 - 4.5)
Age ≥ 24 months	144	35 (24.3)		
Male	100	36 (36.0)	1.6 (1.0 - 2.4)	2.1 (1.1 - 4.0)
Female	114	26 (22.8)		
Weight for height *z *score < -2	28	7 (25.0)	0.8 (0.4 - 1.7)	
Weight for height *z *score ≥ -2	182	54 (29.7)		
Haemoglobin < 11 g/dl	127	45 (35.4)	1.8 (1.1 - 2.9)	1.9 (0.9 - 4.6)
Haemoglobin ≥ 11 g/dl	87	17 (19.5)		
CD4+ < 25%	114	36 (31.6)	1.2 (0.7 - 1.9)	
CD4+ ≥ 25%	85	22 (25.9)		
On HAART	44	10 (22.7)	0.7 (0.4 - 1.3)	
HAART naïve	170	52 (30.6)		
Routine CTX prophylaxis	193	58 (30.1)	1.6 (0.6 - 3.9)	
No routine CTX prophylaxis	21	4 (19.0)		
Previous routine multivitamins	144	44 (30.6)	1.2 (0.7 - 1.9)	
No routine multivitamins	70	18 (25.7)		

^a^Low vitamin B_12 _< 221 pmol/L,

Low folate < 13.4 nmol/L

## Discussion

This paper describes the vitamin B_12 _and folate concentrations of HIV-infected Ugandan children aged 1-5 years and the effect of 2RDA of 14 multiple micronutrients that contained vitamin B_12 _and folate versus the 'standard of care' multivitamins in 1RDA without vitamin B_12 _and folate. Almost a third of the children had low vitamin B_12 _and low folate concentrations at baseline. Very low serum concentrations were uncommon. MMS containing vitamin B_12 _and folate improved both vitamin B_12 _and folate concentrations compared to the 'standard of care' multivitamins.

Vitamin B_12 _and folate concentrations in our study are comparable or slightly lower than what has been reported in children living in other low-income countries [1,2,4,17]. Our findings are also comparable to what other studies in HIV infected adults reported before the HAART era. These studies showed that low vitamin B_12 _concentrations were relatively common with a prevalence ranging between 10 and 35% [18-22].

There are few studies that have examined vitamin B_12 _and folate in HIV infected children in Africa. The prevalence of low vitamin B_12 _concentrations in our study was much higher than the prevalence of 5% reported in South African HIV infected children [23]. Contrary to our findings a study of HIV infected children in New York showed elevated vitamin B_12 _and folate status [24]. Our study included younger children, the majority of whom were symptomatic and not on HAART compared to the New York study. In our study the lack of differences between the HAART and non-HAART stratum in baseline vitamin B_12 _or folate concentrations could be explained by the short duration of HAART compared to other studies.

Twice the recommended dietary allowance of multiple micronutrients improved vitamin B_12 _and folate status compared to the 'standard of care'. This is not surprising since the standard of care supplement did not contain vitamin B_12 _and folate. This implies that the standard of care multivitamin is not enough and many more micronutrients may be required to correct micronutrient deficiencies. A significant number of children still had low concentrations of vitamin B_12 _and folate at the end of 6 months, implying that the duration of supplementation needed to be extended.

Low vitamin B_12 _could be related to folate deficiency or to low vitamin B_12 _binding proteins. Although we did not find an association between low vitamin B_12 _and white blood cell count this does exclude the possibility that low vitamin B_12 _concentrations could be related to neutropenia.

We observed that there was an association between low folate concentrations and low haemoglobin which indicates that the anaemia could partly be attributed to low folate concentrations. In both treatment groups the haemoglobin improved compared to baseline levels. We could not conclusively attribute the anaemia to low folate concentrations since we did not measure red blood cell folate concentrations. Measuring both serum folate and Red cell folate would have yielded more diagnostic information of folate deficiency. However sub-normal serum folate is a useful indicator of folate status that warrants reporting. In a landscape of multiple deficiencies, such as HIV-infected children in a low-income country, there is always the potential for other deficiencies to alter the response, for instance, iron deficiency (the supplement did not contain iron). Folate deficiency has been reported in almost one in two anaemic HIV infected patients [25]. Other authors have reported associations between anaemia, gender and folate deficiency in children whose HIV infection status was not known [26].

Neither MMS nor the 'standard of care' MV improved the immunological status of the study children. In fact there was a slight deterioration in the CD4+ cell counts among the HAART naïve MMS group. This was contrary to findings of a trial in HAART treated HIV infected adults living in the USA where multiple micronutrient supplementation was associated with improved CD4+ cell count compared to a placebo [27]. The lack of effect on CD4+ cell count in our study could be explained by the natural immunological deterioration or it may be apparent due to the small numbers in the HAART group. It is also possible that the supplementation was not long enough to show an impact on CD4+ cell count since one in five children still had low vitamin B_12 _and folate concentrations at 6 months of follow up.

In our setting it is possible that low concentrations of vitamin B_12 _and folate may be related to consumption of marginal or low levels of vitamin B_12 _and folate since twice the recommended dietary intakes increased serum concentrations as opposed to the multivitamin supplement which did not contain vitamin B_12 _or folate. Some authors have shown that Vitamin B_12 _deficiency is rare in HIV infected persons consuming vitamin B_12 _well above the recommended nutrient intakes [28]. In Uganda one third of children aged between 6 and 24 months are likely to be getting diary products in their diet and very few are likely to be getting other animal source foods [29].

We are not certain whether our findings are similar to children in the general population since we had no control group of HIV uninfected children. A study of both HIV-infected and exposed uninfected children in Brazil showed no differences in micronutrient status [30]. However another study indicated that HIV infected children had significantly lower folate levels than the reference children while vitamin B_12 _was similar [31].

We were unable to measure serum homocysteine and methyl malonic acid concentrations which are more reliable indicators of vitamin B_12 _deficiency because of the limited amount of blood that we could draw from the children as we had multiple micronutrients to test for. We also did not conduct absorption studies to examine the impact of malabsorption on baseline micronutrient and post-supplementation status. This paper does not describe the dietary habits of the study children and whether they had an impact on the outcome.

## Conclusion

Low vitamin B_12 _and folate concentrations are common in Ugandan HIV infected children aged 1-5 years. Twice the recommended dietary allowance of 14 multiple micronutrients as opposed to the 6 multivitamin 'standard of care' supplement improved the vitamin B_12 _and folate status of HIV-infected children in Uganda.

## Competing interests

The authors declare that they have no competing interests.

## Authors' contributions

GN participated in the conception, design and implementation of the study, statistical analysis, interpretation and writing of the manuscript. JKT participated in the conception, design and implementation of the study, statistical analysis, interpretation and drafting of the manuscript. CMN participated in the design and implementation of the study. BJB participated in the design, supervised the laboratory work and drafting of the manuscript. TT participated in the conception, design and implementation of the study, statistical analysis, interpretation and drafting of the manuscript. All the authors read and approved the final manuscript.
